# A case report of seizure worsening after perampanel add-on to oxcarbazepine: possible role of metabolic interaction and pharmacogenetics

**DOI:** 10.3389/fpsyt.2025.1621598

**Published:** 2025-09-23

**Authors:** Xin Fang, Menglu Zhu, Lijiang Wang

**Affiliations:** Department of Pharmacy, the Fourth Affiliated Hospital of School of Medicine, and International School of Medicine, International Institutes of Medicine, Zhejiang University, Yiwu, China

**Keywords:** perampanel, oxcarbazepine, combination medication, increased seizures, ethnic factors

## Abstract

**Objective:**

To investigate the factors contributing to increased seizure frequency in patients treated with a combination of oxcarbazepine (OXC) and perampanel.

**Methods:**

The influence of the combined use of perampanel and oxcarbazepine on pharmacokinetics was analyzed, and the results were further confirmed by scoring the Naranjo Adverse Reaction Scale. The influence of race on antiseizure medications (ASMs) was also analyzed.

**Results:**

The concomitant use of perampanel and oxcarbazepine increased the plasma concentration of oxcarbazepine. Scoring with the Naranjo adverse reaction scale indicated that the deterioration of seizures in patients following the addition of perampanel may be associated with the interaction of the two drugs.

**Conclusion:**

The exacerbation of epilepsy in patients may be attributed to the increased blood concentration of oxcarbazepine resulting from its combination with perampanel, which potentially triggers a worsening of seizures. Variations in gene mutations related to Asian factors, as well as differences in drug metabolism enzymes and transporters, may also contribute to alterations in the concentration of ASMs.

## Introduction

**Figure 1:**
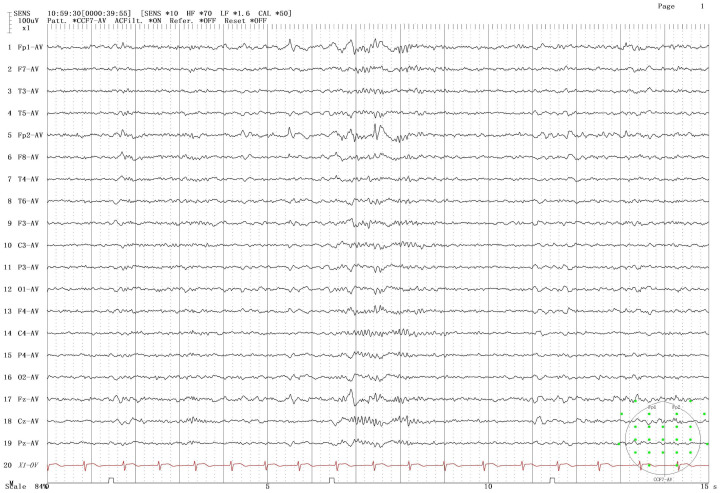
Electroencephalogram (EEG) results of the patient.

The patient was male, 19 years old, and Chinese, with a history of Japanese encephalitis at the age of 3 years, no history of cranial trauma, and no family history of epilepsy. In 2016, the patient presented symptoms such as flexion and convulsion of both upper limbs, gaze on both eyes, and confusion because there was no obvious cause. The patient went to Wenzhou Hospital, Zhejiang, for treatment. According to the patient’s description, the symptoms of the attack were diagnosed as epilepsy, and sodium valproate sustained release tablets were used for treatment. In 2018, owing to poor drug control effects, seizures were still detected when oxcarbazepine was combined with other drugs. After treatment with sodium valproate sustained release tablets combined with oxcarbazepine, there was no recurrence in the subsequent 3 years. Based on such therapeutic effects, the neurologist reduced the dosage of sodium valproate sustained. In June 2023, occasional twitching and stiffness of both upper limbs occurred during the medication reduction period. The neurology department of Wenzhou Hospital chose to maintain the treatment without changing the dosage and continued to observe whether the symptoms worsened before considering changing the treatment plan. On February 28, 2024, the patient came to our hospital for treatment due to the increase in the frequency of epilepsy and was confused during the attack. The patient was 165 cm tall, weighed 60 kg, had a pulse of 80 beats/min, had a blood pressure of 120/80 mmHg, and had a body temperature of 36.5 °C. The patient was awake, developed normally, had good nutrition, and was mentally calm. Laboratory examination revealed the following: neutrophil percentage, 62.7%; hemoglobin, 151 g/L; platelet count, 217*10^9/L; alanine aminotransferase, 9 U/L; aspartate aminotransferase, 15 U/L; total bilirubin, 12 µmol/L; albumin, 51.8 g/L; creatinine, 56 µmol/L; sodium, 140.8 mmol/L; potassium, 4.01 mmol/L; chlorine, 103.6 mmol/L; and calcium, 2.45 mmol/L. The results of routine blood and biochemical tests were basically normal. In addition, long-range video electroencephalogram (EEG) detection revealed a moderate abnormal EEG signal, and the abnormality during onset was an intermittent slow wave with a right frontotemporal area. The sporadic slow waves were sporadic during sleep, and the bilateral temporal area was affected. The EEG results of the patient are shown in **Figure 1**. According to the 2017 ILAE epilepsy classification (1), it is considered focal epilepsy. Our hospital was treated with sodium valproate. In April of the same year, the patient had worsening seizures, a stiff upper limb, and shaking of the lower limbs. The doctors at our hospital used adjunctive treatment of partial-onset seizures with or without secondarily generalized seizures in patients with epilepsy aged 4 years and above according to the indications for perampanel. It is also approved for single-agent treatment of focal seizures in patients aged 4 years and above (2). The combined treatment of perampanel was added. The patient had symptoms such as strong stiffness and shaking about 6 days after the drug was added. The patient had no other abnormal events during the medication period and only took ASMs, which was no different from his previous lifestyle. On the 9th day, he came to our hospital for a follow-up visit, and the treatment with perampanel plus topiramate was stopped. The seizure time, seizure state and clinical treatment are shown in **Table 1**.

**Table 1:** Seizure time, seizure status and clinical treatment of patients.

Seizure time	Seizure status	Clinical treatment
2016	Upper limb convulsions, confusion	Sodium Valproate Sustained Release Tablets (I) 500 mg bid
2018.6.5	Poor seizure control	Sodium valproate sustained-release tablets (I) 500 mg bid+ oxcarbazepine 600 mg bid
2022.11.10	Seizure-free	Sodium valproate sustained-release tablets (I) 250 mg bid+ oxcarbazepine 600 mg bid
2023.6.23	Subjective Electric shock sensation, limb startle, stiffness	Sodium valproate sustained-release tablets (I) 250 mg bid+ oxcarbazepine 600 mg bid
2023.11.10	Transient limb stiffness	Sodium valproate sustained-release tablets (I) 250 mg bid+ oxcarbazepine 600 mg bid
2024.2.28	Confusion	Sodium valproate sustained-release tablets (I) 500 mg in the morning and 250 mg in the evening + oxcarbazepine 600 mg bid
2024.3.28	Transient limb stiffness	Sodium valproate sustained-release tablets (I) 500 mg in the morning and 250 mg in the evening + oxcarbazepine 600 mg bid
2024.4.4	Rigidity of both upper limbs, tremor of lower limbs, gaze of both eyes	Sodium valproate sustained-release tablets (I) 500 mg in the morning and 250 mg in the evening + oxcarbazepine 600 mg bid + perampanel 2 mg qn
2024.4.13	Frequent tonic jitter episodes	Sodium valproate sustained-release tablets (I) 500 mg in the morning and 250 mg in the evening + oxcarbazepine 600 mg bid + topiramate 25 mg bid

## Discussion and analysis

In the US, perampanel is approved for the adjunctive treatment of partial-onset seizures (with or without secondarily generalized seizures) in patients with epilepsy aged 4 years and older and for adjunctive therapy for primary generalized tonic–clonic seizures in patients aged 12 years and older. It is also approved for monotherapy of partial-onset seizures in patients aged 4 years and older.

Glutamate is the main excitatory neurotransmitter in the central nervous system of mammals. The α-amino-3-hydroxy-5-methyl-4-isoxazolepropionicid (AMPA) receptor is the main glutamate-gated ion channel and can mediate most rapid synaptic excitation in the brain (3–5). Impaired regulation of AMPA receptor function can lead to neuronal hyperexcitability and induce epileptic seizures, which makes AMPA receptors potential targets for treatment of epileptic seizures (6). Perampanel is a selective noncompetitive AMPA receptor antagonist that binds noncompetitively to AMPA receptors on the postsynaptic membrane, thereby weakening the excitatory nerve transmission mediated by glutamate, thereby inhibiting epileptic seizures (7, 8). The pharmacokinetics of PER are linear, and it is absorbed quickly after oral administration. The peak time of drug action during fasting is a Tmax of 0.5~2.5 h. Eating does not affect the degree of drug absorption but only slows the speed of drug absorption. The bioavailability is 100%, and the binding rate to plasma protein is 98% (9).

Perampanel is metabolized in the liver, which is mainly mediated by the cytochrome P4503A4 enzyme (CYP3A4) to various metabolites, with a half-life in adults of approximately 105 hours. Perampanel is more sensitive to drug interactions between ASMs. When a cytochrome P450 (CYP) inducer is used simultaneously with perampanel, it reduces the blood concentration of perampanel (10). The interactions between perampanel and other ASMs are shown in **Table 2**.

**Table 2:** The mutual influence of perampanel and ASM concentrations.

ASM combined administration	Effect of ASM on perampanel concentration	Effect of perampanel on ASM concentration
Carbamazepine	Reduced by 2/3	Reduced by <10%
clobazam	No impact	Reduced by <10%
clonazepam	No impact	No impact
Lamotrigine	No impact	Reduced by <10%
Levetiracetam	No impact	No impact
Oxcarbazepine	Reduced by 1/2	Increased by 35%
phenobarbitol	Reduced by 20%	No impact
Phenytoin	Reduced by 1/2	No impact
Topiramate	Reduced by 20%	No impact
valproate	No impact	Reduced by <10%
Zonisamide	No impact	No impact

Moderate and powerful CYP3A4 enzyme inducers, including enzyme-induced ASMs such as oxcarbazepine, can lead to a decrease in plasma concentrations of perampanel. Oxcarbazepine is a new ASM, and its chemical properties are related to those of carbamazepine (11). Carbamazepine has been used for decades and remains the first-line ASM for the treatment of focal epilepsy. However, studies have shown that carbamazepine may aggravate idiopathic generalized epilepsy, especially typical abnormal and myoclonus convulsions (12). Oxcarbazepine is a second-generation structural variant of carbamazepine that was introduced to avoid the formation of 10,11-epoxides to reduce side effects (13).

Oxcarbazepine is a prodrug with a pharmacologically active metabolite of 10-hydroxycarbamazepine. This metabolite exhibits linear pharmacokinetic properties. After oral administration, oxcarbazepine was quickly absorbed, with a Tmax of 10-hydroxycarbamazepine of 3–6 hours. Its bioavailability is 100%, and its volume distribution volume is 0.75 L/kg. The plasma protein binding rate of 10-hydroxycarbamazepine is 40%. Oxcarbazepine is metabolized rapidly and converted into equal amounts of 10-hydroxycarbamazepine (also known as S-licabazepine and monohydroxy derivatives) through cytoplasmic aryl ketone reductase. The conversion from oxcarbazepine to 10-hydroxycarbamazepine is stereoselective, with the concentration of S-enantiomers (with slightly higher pharmacological activity) slightly higher than that of R-enantiomers. 10-Hydroxycarbamazepine is metabolized mainly by glucuronation (51%) and forms dihydroxy metabolites (28%) through hydroxylation of the CYP isozyme. The half-life of 10-hydroxycarbamazepine in adults is 8–15 hours (14, 15).

Studies have shown that taking oxcarbazepine simultaneously can reduce the area under the plasma concentration–time curve of perampanel by 50% (16). According to Patsalos et al., coadministration of oxcarbazepine with perampanel led to a 37% decrease in the mean total plasma concentration of perampanel (17), whereas perampanel simultaneously increased the concentration of oxcarbazepine by approximately 35%. The proposed mechanism underlying this interaction involves perampanel’s selective noncompetitive antagonism of AMPA receptors, coupled with its high protein-binding properties, which may result in competitive binding interactions with other highly protein-bound drugs, significantly affecting their free concentrations (18).

Retrospective clinical studies have shown that oxcarbazepine is related to the exacerbation of epilepsy (19). Philippe et al. reported that six patients with idiopathic generalized epilepsy experienced significant exacerbations, characterized by increased seizure frequency and the emergence of new seizure types following oxcarbazepine treatment (20).

To further explore the correlation between perampanel combination therapy and associated adverse reactions, we utilized the Naranjo adverse reaction scale to assess the association between perampanel and epilepsy exacerbation in this patient cohort (21). As presented in **Table 3**, a Naranjo score exceeding 9 points suggests a positive causal relationship between a drug and adverse drug reactions (ADRs), whereas scores of 5–8 points indicate a probable association, scores of 1–4 points suggest a possible association, and scores of ≤0 points denote doubt. In this case, the Naranjo score related to ADRs from the combination of perampanel was 5 points, indicating a likely association and supporting the conclusion that this combination may have aggravated the patient’s epilepsy.

**Table 3:** Results of the Naranjo scale assessment of seizure exacerbation in patients treated with the perampanel combination.

Scored item	Scores			Reason for rating
	Yes	No	Unknown	
Are there previous conclusive reports on this reaction?		0		There were no reports of seizures aggravated by perampanel combination therapy.
Did the adverse event appear after the suspected drug was administered?	+2			Aggravated seizures occur after the use of perampanel.
Did the adverse reaction improve when the drug was discontinued or a specific antagonist was administered?	+1			The seizures improved after drug withdrawal.
Did the adverse reaction reappear when the drug was readministered?			0	Was not used again.
Are there alternative causes (other than the drug) that could on their own have caused the reaction?		+2		No other seizure induction events occurred when the patient took the drug on time.
Did the reaction reappear when a placebo was given?			0	No placebo applied.
Was the drug detected in the blood (or other fluids) in concentrations known to?			0	No determination of drug concentration taken.
Was the reaction more severe when the dose was increased, or less severed when the dose was decreased?			0	No increase or decrease in dose of perampanel.
Did the patient have a similar reaction to the same or similar drugs in any previous exposure?			0	This patient had not been previously exposed to similar drugs and has not experienced similar reactions.
Was the adverse event confirmed by any objective evidence?			0	No objective evidence.

In addition, the occurrence of adverse reactions caused by ASMs may be related to race. The use of ASMs depend on race, especially in Asia, and the patient in this case was Asian. It is estimated that more than half of the 50 million epilepsy patients worldwide live in Asia. Although Asia has achieved enormous economic development and improvements in health care services, it is a heterogeneous and resource-limited continent. The burden of neuropsychiatric diseases (including epilepsy) is 17.0% greater than that in Africa. In addition, more Asian countries are undergoing epidemiological changes than African countries are, increasing the background risk of chronic diseases such as epilepsy. The main causes of epilepsy are many, such as head injuries, birth trauma, and intracranial infections such as neurocysticercosis or meningoencephalitis. The patient in this case had a history of Japanese encephalitis at the age of 3, which may have been the main cause of his epilepsy. Studies have shown that the region with the largest gap in the treatment of epilepsy is Asia (22), and Asia is also the most populous region in the world. In contrast, poor seizure control can lead to low educational achievement, low employment opportunities and productivity, increased morbidity and mortality, and decreased quality of life. Known genetic factors may play an increasingly important role in epilepsy pathogenesis and the efficacy of ASMs (23). The results of clinical trials conducted in Europe or North American countries do not necessarily apply to Asian populations, and drug responses may vary in Asian populations due to different genetic backgrounds. For example, there are significant racial differences in the degree of liver CYP isoenzyme expression (24), and ASM-induced adverse skin reactions may occur more frequently in the Han and Thai populations (25, 26). People of different ethnic backgrounds may experience changes in the pharmacokinetic (PK) and pharmacodynamic (PD) response of the drug, which may affect dose, efficacy, and safety (27).

Moreover, clinical studies have shown that the efficacy or safety of OXC varies greatly among different populations because of functional changes in metabolic enzymes, transporters and other receptors involved in pharmacokinetics and pharmacodynamics *in vivo*. It has been reported that in Asian epilepsy patients, patients carrying ABCC2c.1249G>A and ABCC2c.-24C>T mutation alleles need higher OXC maintenance doses (28). Ma et al. demonstrated that in Chinese patients with Han epilepsy, the ABCC2c.1249G>AGA genotype was significantly correlated with the OXC maintenance dose. In other words, the OXC maintenance dose required by carriers of mutant alleles is greater than that required by noncarriers (29, 30). In Chinese people, OXC requires a relatively high dose for controlling epilepsy, and the incidence of adverse reactions increases. The worsening of epilepsy in this patient may also be related to the mutational alleles and transporters associated with Asian racial factors.

The exacerbation of epilepsy in this patient may also stem from allele mutations and transporter variations within Asian racial factors. Studies examining the safety profile of perampanel in Asian versus non-Asian populations have revealed differing adverse event profiles. Compared with their non-Asian counterparts, Asian patients reported higher frequencies of irritability, dizziness, lethargy, and headaches at doses of 2 and 4 mg. Conversely, at higher doses (8 and 12 mg), non-Asian patients were more likely to experience fatigue, irritability, dizziness, and headaches than Asian patients were. This discrepancy may be attributed to differences in baseline characteristics between the two populations (31).

The relationship between ASMs and race has an important impact on the safe use of drugs in the clinic. Differences in gene mutations, drug metabolic enzymes, transporters, etc., and racial factors may lead to different responses to ASMs in people of different races. We can choose appropriate drugs on the basis of the patient’s ethnic background, and we can also effectively improve the efficacy and safety of ASMs through genetic testing, personalized dose adjustment and other measures to reduce the risk of side effects.

Epilepsy is a common neurological disease and is a chronic disease caused by highly synchronized discharges of neurons. The causes of its complexity include structure, genetics, infection, metabolism, immunity and other unknown causes (32). Currently, for newly diagnosed epilepsy patients, antiepileptic seizure drugs are still the cornerstone of treatment. Approximately 70% of patients are treated with antiepileptic seizure drugs, and epilepsy can be well controlled (33). However, approximately 30% of patients do not respond to one or more ASMs, whose epilepsy is considered drug resistant (34) and usually requires combined treatment with ASMs.

Sodium valproate (VPA) is a mature and widely used ASM on the market for the treatment of generalized and focal epilepsy in adults and children (35). The pharmacological effects of sodium valproate include enhancing the transmission of γ-aminobutyric acid (GABA) energy, reducing the effects of excitatory amino acids, blocking voltage-gated sodium channels, and regulating dopaminergic and serotonergic transmission (36). The most common adverse reactions to sodium valproate include sedation, fatigue, tremor, gastrointestinal symptoms, weight gain, etc. (37). In addition, an important advantage of valproate is that it can be used in different dosage forms for parenteral or oral use. Sustained release formulations of sodium valproate are ideal for minimizing serum drug fluctuations and can be administered once or twice a day. Sodium valproate remains the preferred choice for almost all types of generalized epilepsy (38).

Oxcarbazepine is a commonly used ASM with a chemical structure similar to that of carbamazepine (11). The drug has been approved by the U.S. Food and Drug Administration for partial and generalized antonymosis paroxysmal seizures in adults and pediatric patients. It prevents seizures mainly by blocking voltage-dependent sodium channels, similar to carbamazepine (15). Because of its comparable effectiveness but better safety and tolerance, oxcarbazepine is often used as a carbamazepine alternative in patients who cannot tolerate carbamazepine (39).

Perampanel is the first and only noncompetitive AMPA receptor antagonist approved by the FDA; it binds noncompetitively to AMPA receptors on the postsynaptic membrane, thereby inhibiting glutamate-induced hyperneurotransmission and exerting an antiepileptic effect (7, 8). AMPA receptors are widely distributed in the central nervous system and are present in all areas associated with epilepsy. Increased AMPA receptor expression may be a common endophenotype in epilepsy; therefore, AMPA receptor antagonists have potential as broad-spectrum antiepileptic agents. Many animal experiments have confirmed that perampanel has broad-spectrum anti-attack activity. As a noncompetitive AMPA receptor antagonist, perampanel has new mechanisms of action, and it has good drug tolerance for both additive treatment and monotherapy.

Topiramate (TPM) was approved in the United States in 1996 as a newer ASM (40), a broad-spectrum ASM that has been authorized for single-agent therapy and adjuvant treatment of multiple types of epilepsy, including focal epilepsy, generalized tonic–clonic seizures (GTCSs), adolescent myoclonus epilepsy (JME), epileptic encephalopathy (such as Webster’s syndrome, Dravt syndrome and Ronx–Gastau syndrome (LGS)) and state epilepsy (SE) in children and adults (41). The basic mechanisms of the antiepileptic effect of topiramate (TPM) include blockade of voltage-dependent sodium channels and kainate-induced currents, enhanced γ-aminobutyric acid (GABA)-induced current reactions, effects on voltage-activated calcium ion channels, inhibition of carbonic anhydrase isozymes, and interactions with protein kinase phosphorylation sites (42). Topiramate (TPM) can also cause several adverse reactions, including drowsiness, dizziness, fatigue, insomnia and weight loss (43). In recent years, adverse reactions to TPM, especially cognitive impairment, have attracted increasing attention (44).

Drug treatment is still the main treatment method for epilepsy, but no single drug is suitable for all environments and populations. Therefore, by systematically comparing the differences in the efficacy, safety, and tolerance of different ASMs, we provide clinicians with evidence-based and visual decision-making methods so that they can quickly lock or adjust the optimal treatment plan on the basis of the patient’s seizure type and individual characteristics, thereby improving efficacy, reducing risks and improving long-term compliance.

## Conclusion

This case report examines the adverse reactions of exacerbated epilepsy associated with the combination of perampanel and oxcarbazepine. This phenomenon may be attributed to the elevated concentration of oxcarbazepine following the coadministration of these two drugs. The underlying interaction mechanism may involve the high protein-binding affinity of perampanel, which could diminish the binding capacity of oxcarbazepine to plasma proteins, subsequently increasing its concentration in circulation.

However, this study has many limitations. The number of cases in this paper is very small, with only one report; coupling cannot be ruled out, and the blood concentrations of perampanel and oxcarbazepine have not been determined simultaneously. Moreover, there was no control group, so the impact of racial differences on drug concentrations could not be quantified. In the future, we can design multicenter, prospective cohorts or randomized cross-examinations to systematically compare the pharmacokinetic/pharmacodynamic differences in Asian and European populations in the combined regimen of perampanel and oxcarbazepine, actively explore the feasibility of binding competition and free concentration monitoring of perampanel and oxcarbazepine proteins, and provide a laboratory basis for individualized drug delivery. When a combination regimen of perampanel and oxcarbazepine is used in Asian patients in clinical practice, a low dose should be used, and the frequency of attacks and adverse reactions should be monitored within 1–2 weeks. If the attack worsens, priority will be given to the detection of free oxcarbazepine concentrations and liver and renal function, and the dosage of oxcarbazepine should be lowered if necessary.

Additionally, we explored the relationship between antiepileptic medications and Asian populations, noting that genetic mutations, variations in drug-metabolizing enzymes, transporters, and other ethnic differences may significantly influence the concentration of ASMs.

This underscores the importance of considering the safety of combination therapies and the need for personalized medication approaches tailored to specific racial and ethnic groups in the clinical application of ASMs.

## Data Availability

The original contributions presented in the study are included in the article/supplementary material. Further inquiries can be directed to the corresponding author/s.
